#  Ankyloblepharon Filiforme Adnatum in a New Born 

**Published:** 2016-01-01

**Authors:** Pratap Patra, Aastha Singh

**Affiliations:** 1 Department of Pediatrics, All India Institute of Medical Sciences, Patna, Bihar, India; 2 Department of Ophthalmology, All India Institute of Medical Sciences, Patna, Bihar, India

**Dear Sir**

A 6 day old male baby born to second gravida mother presented with complains of rapid breathing for two days. On examination he was lethargic, heart rate was 154/minute, respiratory rate 64/minute, temperature was 36.40C. He had cleft lip and cleft palate and ophthalmic examination revealed a single central extensile thin fibrous adhesion between the eyelid margins at the grey line of the left eye (Fig. 1). He was screened for late onset neonatal sepsis and started on cefotaxime and amikacin. Septic screen parameters were negative. His general condition and vitals improved after 72 hours of hospitalisation. The adhesion was divided with Castroviejo corneoscleral scissors by cutting at its base, flush with the lid margin. The adhesion as separated first from the upper lid margin followed by the lower lid margin. After the adhesion was removed reassessment of the eye was done. Ocular motility, papillary reactions, anterior segment and fundus examinations were within normal limits. His ophthalmological condition was normal during follow-up in well baby clinic.

**Figure:**
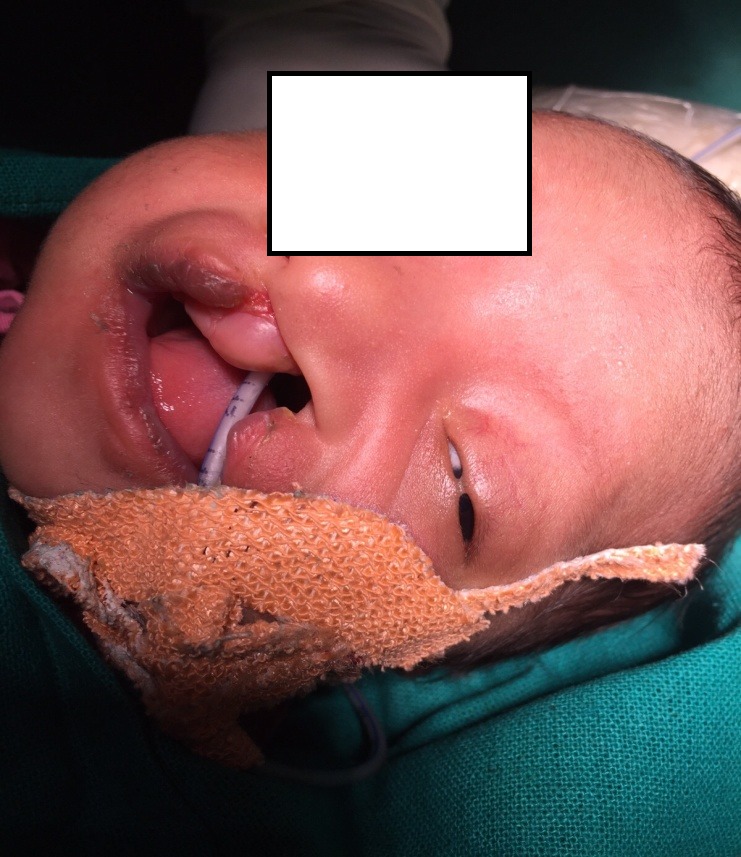
Figure 1: Left eye shows an adhesion between eye lids.

Ankyloblepharon filiforme adnatum (AFA) is a rare benign congenital anomaly of the eye lid and was first described by Von Hasner in 1881. It is characterized by full thickness fusion of eyelid margins by single or multiple fine bands of expansible tissue connecting the lid margins at the grey line. The developing eyelids remain fused until the fifth month of gestation after which they begin to separate to organize into an upper and lower lid. This separation is usually complete by seventh month of gestational age and thus Ankyloblepharon is an unusual finding at birth. It is a solitary malformation which occur sporadically but, in most familial cases it occurs together with cleft lip and cleft palate as an autosomal condition in otherwise a healthy relative (MIM 106250) [1]. Rosenman et al have divided AFA into four subgroups [2]. Group 1 is not associated with any other condition where as group II is associated with central nervous system and cardiovascular malformation. Group III is associated with ectodermal dysplasia. The index case falls in group IV, which is associated with cleft lip and cleft palate. Baca et al has proposed a fifth group which is associated with chromosomal anomaly [3]. Additionally, it may be associated with other congenital anomalies e.g. hydrocephalus, meningocele, imperforate anus, bilateral syndactyly, infantile glaucoma and cardiac condition such as patent ductus arteriosus and ventricular septal defect [4]. The index case had no other congenital anomalies.

Though exact etiology is not known, it is thought to be due to interplay of temporary epithelial arrest and rapid mesenchymal proliferation which allows union of eye lid at abnormal position [5]. Treatment of AFA is very simple and the elastic bands can be divided by using suture cutter. Sedation or local anaesthesia is not needed.

## Footnotes

**Source of Support:** Nil

**Conflict of Interest:** None
